# Genomic Characterization of the Index Case of Human Monkeypox Virus Infection in Mali, 2025

**DOI:** 10.3390/v18030294

**Published:** 2026-02-28

**Authors:** Noumou Yakhouba Keita, Mahamadou Abdou, Mohamed Ag Baraïka, Klema Marcel Kone, Ousmane Kamena, Elysabette Coulibaly, Mariam Sama Sangare, Korika Diakite, Dorcas Waruguru Wanjohi, Boubacar Doumbia, Harris Onywera, Moussa Moise Diagne, Ibrehima Guindo

**Affiliations:** 1 Institut National de Santé Pulique, Hippodrome Route de Koulikoro Rue 235 Porte 52, Bamako BP 1771, Mali; mohamediallo1984@yahoo.fr (M.A.); moagba08@yahoo.fr (M.A.B.); kklema3@gmail.com (K.M.K.); ousmanekamena9@gmail.com (O.K.); coulsabeth@gmail.com (E.C.); sangaremariamsama152@gmail.com (M.S.S.); korikad10@gmail.com (K.D.); doumbiab44@gmail.com (B.D.); guindo50@gmail.com (I.G.); 2 Faculty of Pharmacy, Université des Sciences des Techniques et des Technologies de Bamako, Point G Campus, Bamako BP 1805, Mali; 3 Africa Centres for Disease Control and Prevention (Africa CDC), Addis Ababa P.O. Box 3243, Ethiopia; dorcasw@africacdc.org (D.W.W.); onywerad@africacdc.org (H.O.); 4 Institut Pasteur de Dakar, Dakar BP 220, Senegal; moussamoise.diagne@pasteur.sn

**Keywords:** monkeypox virus, Mpox, genome, Mali, index, outbreak, emerging

## Abstract

Mpox is a zoonosis caused by the monkeypox virus. Here, we report Mali’s index Mpox case, which was clinically identified at the Mali–Guinea border by the national telemedicine center and confirmed by PCR. The library prepared with NextGenPCR™ MPXV Sequencing Library Prep and sequenced on Minion MK1C revealed a genome length of 197,122 bp with an average depth of 1284.4×. The strain belonged to Clade IIb G1 lineage and exhibited 85 mutations relative to NC_063383.1. To decipher genomic epidemiology, genomes ≥ 195 kb were retrieved from NCBI and aligned with MAFFT. Time-resolved phylogenetic reconstruction and ancestral trait inference were performed with TreeTime v0.11.4. A median joining network was built with Popart v1.7. Phylogeographic analysis revealed clustering with Clade IIb (G.1 lineage) linked to the May 2025 outbreak in Sierra Leone.

## 1. Introduction

Monkeypox virus (MPXV), which causes Mpox, a member of the Orthopoxvirus genus, is an enveloped double-stranded DNA virus endemic in multiple African countries [1]. In 2022, it attracted global attention due to a widespread outbreak in non-endemic regions. Mpox typically presents itself as a febrile disease associated with skin and/or mucosal rash, with a broad spectrum of clinical severity [2].

The genomic linear DNA is approximately 197 kilobase (kb) characterized by a central conserved region flanked by roughly 6.4 kb inverted terminal repeats (ITRs). Proteins encoded by genes in the conserved central region of the genome are involved in transcription, replication and assembly while the ITRs are thought to be involved in pathogenesis and host range [3]. MPXV is genetically classified into clade I and clade II. Clade I, formerly known as the Congo Basin clade, associated with severe clinical symptoms and substantial mortality (4–11%), is endemic to central Africa, notably the Democratic Republic of Congo. Clade II, previously known as the West African clade, associated with mild clinical presentations and lower mortality (4%), was geographically confined until the global circulation of 2022 [4]. Clade Ib first emerged in September 2023 in South Kivu (DRC), followed by an upsurge in East Africa that has spread to several African regions [5].

The emergence of the Clade II B.1 lineage in 2022 expanded global circulation, with multiple countries reporting Mpox cases for the first time, warranting the declaration of Mpox as a public health emergency of international concern (PHEIC) by the WHO in 2022. The upsurge in Mpox cases in 2024 warranted the declaration of Mpox as a public health emergency of continental concern by the Africa CDC in 2024, followed by another PHEIC declaration by the WHO the same year [6,7].

Since 2022, 177,750 laboratory-confirmed cases have been detected in 143 countries, with 477 associated deaths. Regionally, in 2025, multiple West African countries registered a surge in cases. Sierra Leone reported 5442 by 26 October, despite reporting none in 2024. Guinea, a neighboring country to Mali, went from 2 cases in 2024 to 1169 cases by 9 November 2025. Senegal and Cote d’Ivoire, both of which neighbor Mali, also reported cases, with Senegal confirming circulation of both Clade Ib and Clade IIb [8]. In Mali, all suspected cases tested negative (by real-time PCR) until 14 November 2025, when the index case was confirmed. Genomic characterization and rapid data sharing are crucial in tracking viral evolution and transmission dynamics and informing public health decisions. We describe the clinical, molecular, and phylogeographic features of the index Mpox case in Mali.

## 2. Case Description

### 2.1. Location

Kourémalé, situated 125 km from Bamako, is a border community with Guinea where socioeconomic activities are dominated by artisanal gold mining.

### 2.2. Clinical Presentation

On 14 November 2025, a 35-year-old male from Burkina Faso working in artisanal gold mining and frequently traveling between Burkina Faso, Mali and Guinea presented to a district health center in Kourémalé, at the Mali–Guinea border, with fever and a vesiculopustular rash that began on 31 October 2025. Lesions were mainly located on the face and genital region. Initially diagnosed with dermatosis and treated with topical antiseptics, he was later evaluated through the national telemedicine program because of persistent fever. A clinical diagnosis of Mpox was made, and acyclovir with supportive care was initiated, resulting in favorable clinical improvement before transfer to the infectious diseases department of CHU du Point G.

### 2.3. Epidemiological Investigation

Investigators identified 5 direct contacts (including the nurse and a neighbour) and 43 indirect contacts. A swab was collected from the nurse. Contact tracing is underway to identify and isolate all contacts and risk communication and community engagement strategies are being implemented to help communities understand Mpox risks, adopt preventive measures, and support the overall outbreak response.

### 2.4. Molecular Diagnosis, Genomic Characterization and Transmission Dynamic

All laboratory activities were performed at biosafety level 2 while strictly adhering to biosafety and biosecurity measures. Blood and swab samples collected on 14 November were subjected to DNA extraction using the Qiagen DNA Mini Kit (Qiagen, Hilden, Germany) and Mpox detection performed by real-time PCR with LightMix^®^ Modular Monkeypox on LightCycler 480 II (Roche, Berlin, Germany) following the manufacturer’s instructions. The library was prepared with the NextGenPCR™ MPXV Sequencing Library Prep (NextGenPCR, Goes, The Netherlands) as previously described [9]. The library containing the index specimen in duplicate and a negative control was barcoded with the Rapid Barcoding Kit 24 V14 (Oxford Nanopore Technology, Oxford, UK), loaded onto a MinION MK1C running Miniknow v25.05.12, and sequenced for approximately 5 h. High-accuracy basecalling was performed with Dorado v7.9.8.

After sequencing, adapters were removed with porechop v0.2.4 and reads were quality-filtered with fastp v1.0.1. The resulting reads were aligned to the MPXV reference genome (NC_063383.1) with minimap2 2.30-r1287, and consensus sequences were generated with samtools v1.22. Clade and lineage assignments were obtained using Nextclade v3.18.0 [10,11].

For transmission dynamic studies, all MPXV genomes collected between 1 January and 22 November 2025 on NCBI were downloaded and filtered for a genome length ≥ 195 kb. Burkina Faso, despite the few suspected cases, had not reported any confirmed case by the end of 2025. The sequences were aligned with MAFFT v7.526 while phylodynamic analysis was performed using Augur toolkit v31.3.0 [12]. The build was visualized on Auspice v2.67 [13]. To assess the genetic relationship with regional strain, pairwise nucleotide identity was calculated with the identity by alignment method. Identity score was defined as

(1) $$\mathrm{identity}=\frac{\mathrm{matches}}{\mathrm{alignment}\;\mathrm{length}-\mathrm{gaps}}\times 100$$

To resolve fine-scale mutational distance, a median-Jjoining network was constructed on Population Analysis with Reticulate Trees (Popart) v1.7 [14]. Mutational steps were quantified with hatch marks on network branches.

## 3. Results

To decipher molecular epidemiology of the Mpox case, genomic sequencing was performed. A genome with a length of 197,122 bp was obtained with 99.8% coverage with an average depth of 1284.4×. The strain, named Mpx-1-11-14-25-NPHI-ml, belonged to clade IIb G.1 lineage, with 85 mutations relative to NC_063383.1 (Nigeria, 2018). The Mali genome (Mpx-1-11-14-25-NPHI-ml; BioProject PRJNA1367904) is closely related to strains that circulated in Sierra Leone and Guinea in May and June 2025, respectively, with 99.889% and 99.947% identity. The envelope (OGP57) and surface glycoprotein (OGP210) are highly conserved across the three genomes (Table 1).

The Mali genome (Mpx-1-11-14-25-NPHI-ml; BioProject PRJNA1367904) exhibited several distinguishing features relative to the closest contemporaneous Sierra Leone strains. These included five unique nucleotide substitutions (C75602A, C84548T, C134460T, C152336T, and C174267T), two homoplastic substitutions (G2591A and C194619T), and three non-synonymous substitutions affecting OPG002 (S54F), OPG096 (D6N), and OPG002_dup (S54F). The OPG002 gene also known as J2L is the Ortholog of VACV-Cop C22L. Located in the inverted terminal repeat, this gene encodes Cytokine response-modifying protein B (CrmB), which acts as a soluble decoy receptor for tumor necrosis factor (TNF) [15]. In addition, three short gaps totaling 4 bp were detected. The mutation spectrum was dominated by C → T and G → A transitions, consistent with host apolipoprotein B mRNA editing catalytic polypeptide-like 3 (APOBEC3)-associated mutational signatures.

**Figure 1:**
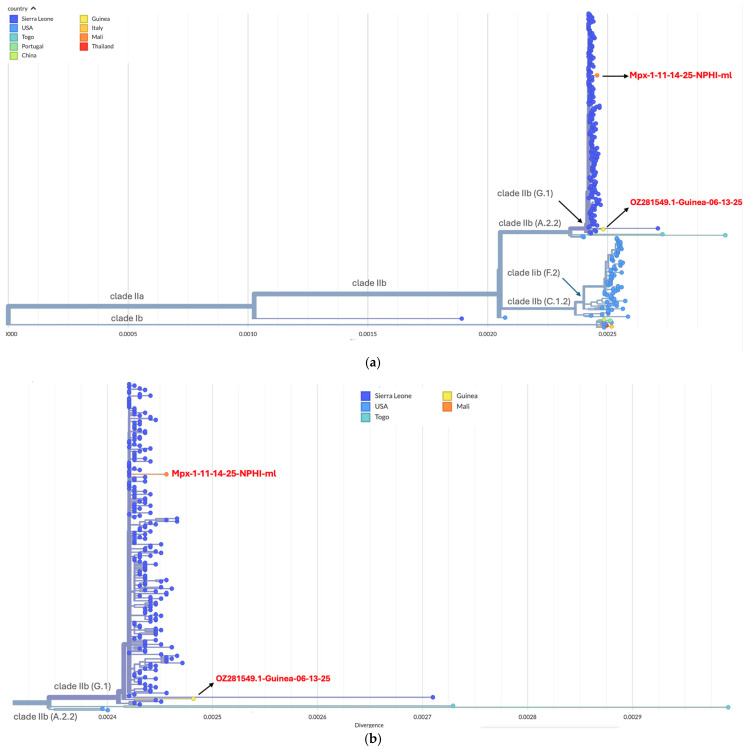
Phylogenetic tree depicting clade and lineage evolution. (**a**) Phylogenetic placement of the Malian strain. (**b**) Subphylogenetic tree of G.1 lineage. Mpx-1-11-14-25-NPHI-ml (highlighted) nests within the G.1 lineage (ancestral anchor: Sierra Leone OZ271447.2). Branch lengths represent nucleotide substitutions per site. The distinct branching of the Mali isolate, characterized by five private SNPs.

Phylogenetically, Mpx-1-11-14-25-NPHI-ml clustered within the clade IIb G.1 lineage and branched from the Sierra Leone clusters detected in May 2025, showing close genetic relatedness to the limited sequences available from Guinea (Figure 1).

**Figure 2:**
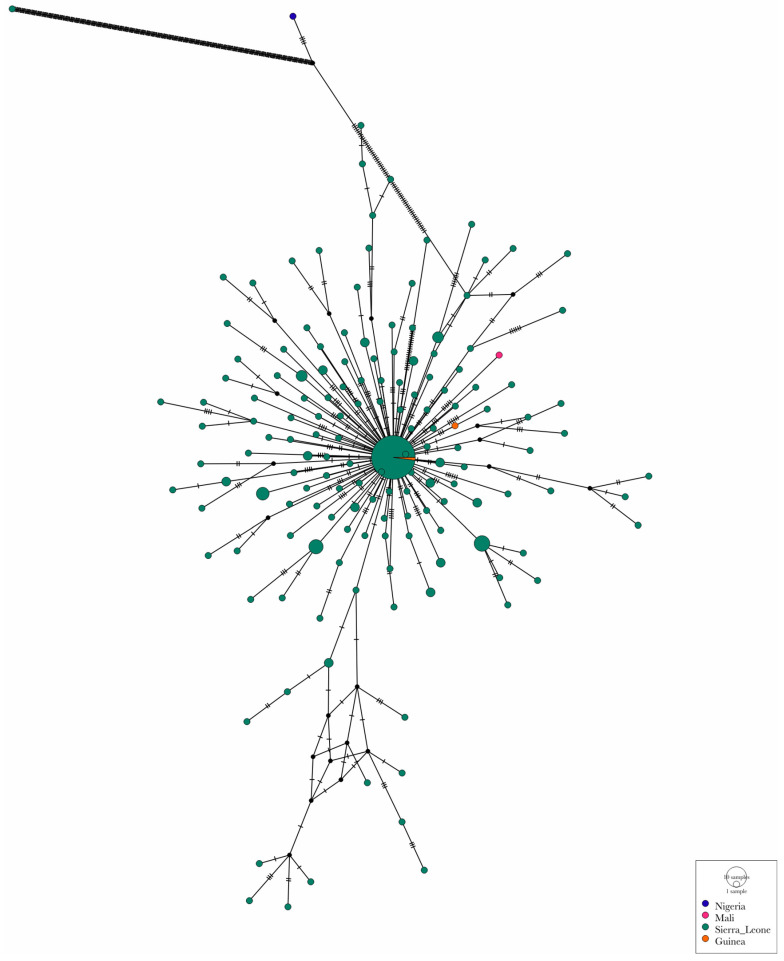
Median joining network of the 2025 West African Mpox G.1 lineage outbreak. The network illustrates the genetic relationships between the Malian index case and regional isolates. Each circle (node) represents a different haplotype geographically color-coded. The central hub (haplotype OZ346733.1) represents the dominant Sierra Leone haplotype from which regional lineages disseminated in a star-like expansion. Hatch marks on branches represent the number of nucleotide substitutions between haplotypes.

Both the Malian and Guinean strain are closely related to the strain responsible for the outbreak in Sierra Leone in May 2025. The median joining network revealed a star-like diversification from a central dominant haplotype (OZ346773.1, Sierra Leone). The lineage progressed from the central haplotype to a Guinea-specific node through five mutation steps. Interestingly, a Sierra Leone node was identified as a terminal descendant of the branch. In contrast, the Malian isolate remains a different satellite node. While it is rooted in the same central Sierra Leonean haplotype, it has accumulated six unique mutations, distinguishing it from the central outbreak hub and the shared Guinea–Sierra Leone haplotype (Figure 2).

**Figure 3:**
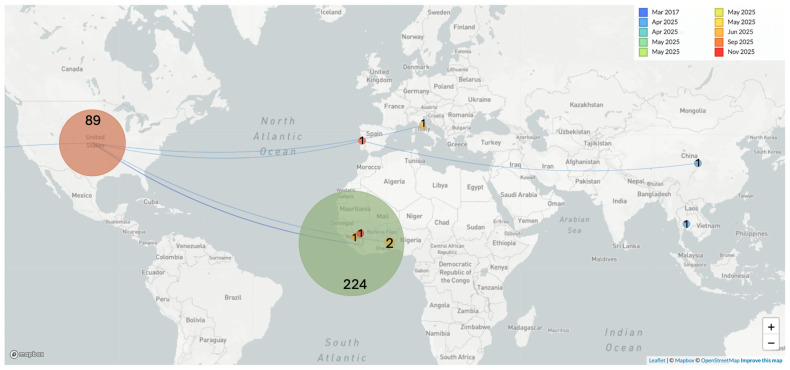
Spatiotemporal circulation of MPXV in 2025.

Spatiotemporal reconstruction suggests that the A.2.2 lineage may have been introduced into Sierra Leone from the USA, as previously reported [11], before giving rise to the G.1 lineage that later circulated in both Guinea and Sierra Leone (Figure 3).

Deme (circle) diameter is proportional to the number of cases with the corresponding number given in each circle. The transmission line is colored by date and proportional to thickness. Thick lines represent more observed case movement between two locations. Transmission direction is inferred by the orientation of the arc; from source to origin location, the arc is positively oriented. That is, the curve is on the left side. From origin to source, the arc is negatively oriented.

## 4. Discussion

The genomic characterization of the Mpx-1-11-14-25-NPHI-ml strain provides critical insight into the ongoing diversification of Clade IIb, lineage G1 within western Africa. With a high depth assembly (1284.4×) and 99.8% coverage, this genome represents a robust reference for monitoring the molecular epidemiology of Mpox in Mali.

The mutation profile of the index strain, consisting of 85 mutations relative to the reference NC_063383.1, is characterized by a high frequency of C → T and G → A transitions, consistent with host-mediated viral editing enzymes. The dominance of these transitions aligns with the evolutionary trajectory observed globally since the 2022 multi-country outbreak, confirming that MPXV continues to undergo accelerated evolution driven by the human host immune system rather than standard viral polymerase errors [16].

While the strain is phylogenetically linked to contemporaneous Sierra Leone strains, the presence of five unique mutations (C75602A, C84548T, C134460T, C152336T, and C174267T) and two homoplastic mutations (G2591A and C194619T) suggests independent evolutionary pressure. Using a median-joining network to resolve fine-scale relationships, we identified a central Sierra Leone hub (OZ346773.1) as the primary source of regional dissemination. The discovery of a shared haplotype between Guinea and Sierra Leone separated by zero mutational steps provides empirical evidence for viral dissemination across borders. This finding is consistent with high human mobility facilitated by regional trades including gold. In contrast, the Malian introduction presents a distinct genomic signature. The absence of shared mutations between the Malian isolate and the shared Guinea–Sierra Leone lineage indicate that the virus likely traveled on an independent path through the region. Overall, the West African wave was not a single uniform wave but rather a complex series of divergent transmission chains moving simultaneously.

The identification of non-synonymous substitutions in specific open reading frames warrants further investigation. In fact, S54F substitution in OPG002 and its duplicate OPG002_dup is of particular interest as it is situated in the ITR and acts as a TNF-decoy receptor to the host immune response [15]. On the other hand, OPG096 is well conserved in the gene in the Orthopoxvirus genus. In the Vaccina virus, OPG096 is homologous to A12L (following VACV-Cop nomenclature). It is involved in the proteolytic processing of major core proteins. The D6N (aspartic acid to asparagine) mutation is in the N-terminus of the protein. While it is a conservative mutation (both amino acids are polar), mutations in the core structural proteins such as OPG092 could potentially impact the efficiency of virion assembly.

## 5. Conclusions

Genomic data confirmed the first human Mpox case in Mali, highlighting the value of the telemedicine program in extending health coverage and improving surveillance sensitivity. Timely sharing of genomic data enabled reconstruction of spatiotemporal transmission patterns, revealing multi-country transmission fueling the emergence of new lineages. The analysis confirms the presence of an evolving G.1 lineage in Mali, characterized by distinct features and a strong APOBEC3 mutational signature. The unique non-synonymous mutation in immune modulating (OPG002) and structural (OPG096) proteins highlight the necessity of ongoing functional studies to determine if these substitutions correlate with altered virulence and transmissibility. The identification of a 6-substitution mutational distance between the Malian isolate and the central Sierra Leonean hub alongside a shared haplotype between Guinea and Sierra Leone underscore the high degree of regional connectivity and rapid viral evolution. Integration of national telemedicine into epidemiological surveillance, active cross-border surveillance, risk communication and genomic characterization is essential in controlling viral spread and burden. Sustaining genomic capacity remains essential for tracking viral evolution and informing timely public health interventions.

## Figures and Tables

**Table 1:** Identity score of the Malian strain relative to contemporaneous strain.

Sequence ID	Origin	Date (mm-dd-yyyy)	Genome Length (bp)	% Identity to Mali	Phylogenetic Status
Mpx-1-11-14-25-NPHI-ml	Mali	11-14-2025	197,122	100%	Index case (G.1)
OZ281549.1	Guinea	06-13-2025	197,487	99.947%	Regional relative (G.1)
OZ261647.2	Sierra Leone	01-10-2025	197,178	99.887%	Lineage ancestor (G.1)
OZ271447.2	Sierra Leone	05-31-2025	197,180	99.889%	Regional relative (G.1)
OZ346773.1	Sierra Leone	05-12-2025	197,184	99.818%	Central haplotype (G.1)
NC_063383	Nigeria	08-24-2018	197,209	99.850%	Global reference

## Data Availability

The sequence reads archived are available under the bioproject accession PRJNA1367904.

## Supplementary Materials

The following supporting information can be downloaded at https://www.mdpi.com/article/10.3390/v18030294/s1, S1: Nexstrain build in json format: S1_Mali_Mpox-index-case.json. S2: accession number and associated metadata used for phylogenetic and transmission dynamic: S2_auspice_final_filtered_metadata.tsv.

## Abbreviations

The following abbreviations are used in this manuscript:

APOBEC3	Apolipoprotein B mRNA editing catalytic polypeptide-like 3
CDC	Centres for Diseases Control and Prevention
*CrmB*	Cytokine response-modifying protein B
DNA	Deoxyribonucleic acid
FHI	Family Health International
INSP	Institut national de santé publique
IPD	Institut Pasteur de Dakar
ITR	Inverted terminal repeat
MAFFT	Multiple Alignment using Fast Fourier Transform
MPXV	Monkeypox virus
NCBI	National Center for Biotechnology Information
PCR	Polymerase chain reaction
PGI	Pathogen genomics initiative
TNF	Tumor necrosis factor
WHO	World Health Organization
